# Targeting Middle East Respiratory Syndrome Coronavirus Spike Fusion Machinery With Antiviral Peptides: In Silico Exploration of the Heptad Repeat 2 Domain

**DOI:** 10.1002/mbo3.70299

**Published:** 2026-05-04

**Authors:** Nasser Alotaiq, Doni Dermawan, Samir Chtita

**Affiliations:** ^1^ Health Sciences Research Center (HSRC) Imam Mohammad Ibn Saud Islamic University (IMSIU) Riyadh Saudi Arabia; ^2^ Department of Applied Biotechnology, Faculty of Chemistry Warsaw University of Technology Warsaw Poland; ^3^ Laboratory of Analytical and Molecular Chemistry, Faculty of Sciences Ben M'Sik Hassan II University of Casablanca Casablanca Morocco

**Keywords:** antiviral peptide, heptad repeat 2, MERS‐CoV, molecular dynamics simulation, peptide‐protein interactions

## Abstract

Middle East Respiratory Syndrome Coronavirus (MERS‐CoV) remains a significant global health threat, necessitating the development of effective antiviral therapeutics. Targeting the heptad repeat 2 (HR2) domain of the MERS‐CoV spike protein offers a promising strategy to inhibit viral fusion and entry into host cells. This study investigates a panel of antiviral peptides (AVPs), focusing on Griffithsin, Brevinin‐2, and CCL20, to evaluate their potential as fusion inhibitors against the HR2 domain. Employing comprehensive computational approaches, including molecular docking, molecular dynamics (MD) simulations, and MM/PBSA binding free energy calculations, we characterized the peptide‐protein interactions and stability of these AVPs in complex with HR2. Our results demonstrate that Griffithsin, Brevinin‐2, and CCL20 exhibit stronger binding affinities (− 213.69, −168.83, and −165.17 kcal/mol, respectively) compared to the standard inhibitor Peptide‐6 (− 49.73 kcal/mol). MD simulations reveal stable complexes and indicate disruption of critical hydrogen bonds in the Ile1255–Gln1271 region of HR2, essential for six‐helix bundle formation and viral fusion. Physicochemical analyses further suggest favorable stability, half‐life, and low hemolytic potential, supporting their suitability as therapeutic candidates. These findings align with prior studies highlighting the broad‐spectrum antiviral activity of Griffithsin and validate the therapeutic promise of Brevinin‐2 and CCL20. While this computational investigation lays the groundwork, further *in vitro* and *in vivo* validation and optimization of pharmacokinetics and delivery are necessary for clinical development. This study advances the rational design of peptide‐based fusion inhibitors targeting MERS‐CoV and provides valuable insights into antiviral strategies against emerging coronaviruses.

## Graphical abstract

Griffithsin, Brevinin‐2, and CCL20 were identified as potent MERS‐CoV fusion inhibitor candidates targeting the HR2 domain through integrated molecular docking, MD simulations, and MM/PBSA analyses. These peptides demonstrated superior binding stability and favorable safety profiles compared to the standard inhibitor, supporting their potential as antiviral therapeutics.

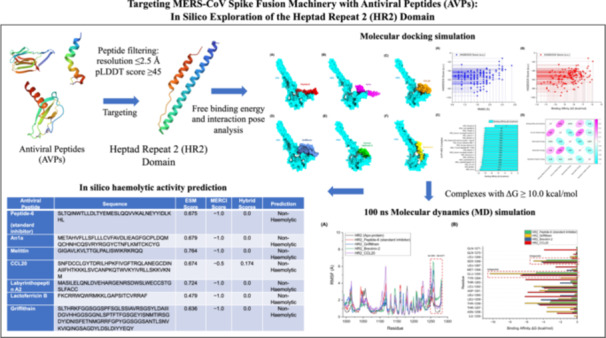

**Abbreviations**
6‐HB: six‐helix bundle
APD3: antimicrobial peptide database 3
CASTpFold: computed atlas of surface topography of the universe of protein folds
DPP4: dipeptidyl peptidase 4
GRAVY: grand average of hydropathicity
HADDOCK: high ambiguity driven protein‐protein docking
HD5: human defensin 5
HR1: heptad repeat 1
HR2: heptad repeat 2
ICs: intermolecular contacts
MD: molecular dynamics
MERCI: motif‐emerging and with classes‐identification
MERS‐CoV: Middle East respiratory syndrome coronavirus
ML: machine learning
MM/PBSA: molecular mechanics/Poisson‐Boltzmann surface area
NIS: non‐interacting surface areas
NPT: number of particles, pressure, and temperature
NVT: number of particles, volume, and temperature
OPLS‐AA/L: optimized potentials for liquid simulations
pI: isoelectric point
pLDDT: predicted local distance difference test
PME: particle mesh Ewald
PRODIGY: PROtein binDIng enerGY prediction
RBD: receptor‐binding domain
RMSD: root mean square deviation
RMSF: root mean square fluctuation
RoG: radius of gyration
SPCE: single point charge extended

## 1 Background

The emergence of Middle East Respiratory Syndrome Coronavirus (MERS‐CoV) in 2012 marked a significant addition to the list of zoonotic pathogens with pandemic potential (Fehr et al. 2017). First identified in Saudi Arabia, MERS‐CoV has since caused sporadic outbreaks with a high case fatality rate, estimated at approximately 43% (Al‐Raddadi et al. 2020). Despite global surveillance and containment efforts, the absence of approved vaccines or specific antiviral therapies underscores the urgent need for effective therapeutic strategies against MERS‐CoV infections (Q. Wang et al. 2016). Currently, clinical management of MERS‐CoV infection relies primarily on supportive care, including oxygen supplementation, mechanical ventilation for severe respiratory failure, hemodynamic stabilization, and management of secondary bacterial infections (Albarrak et al. 2019; Dyall et al. 2017). In some cases, repurposed or investigational agents such as ribavirin, interferon‐α/β, lopinavir/ritonavir, and corticosteroids have been administered; however, clinical outcomes remain inconsistent, and none of these approaches have demonstrated definitive efficacy in controlled trials (Kim et al. 2016). Consequently, there remains an unmet need for targeted antiviral strategies that directly inhibit viral entry and replication. MERS‐CoV is an enveloped, positive‐sense single‐stranded RNA virus belonging to the Betacoronavirus genus (Goyal et al. 2022). Its spike (S) glycoprotein, a trimeric class I fusion protein, facilitates viral entry into host cells and is divided into two functional subunits: S1, responsible for receptor binding, and S2, which mediates membrane fusion (Abdelrahman et al. 2020; Li et al. 2019). The S1 subunit contains the receptor‐binding domain (RBD) that specifically interacts with the human dipeptidyl peptidase 4 (DPP4) receptor, initiating viral attachment. Subsequent conformational changes in the S2 subunit lead to the fusion of viral and host membranes, a critical step for viral entry and replication (Bassendine et al. 2020; Yuan and Wen 2021).

Two heptad repeat regions within the S2 subunit are central to the membrane fusion process: heptad repeat 1 (HR1) and heptad repeat 2 (HR2) (Forni et al. 2015). HR1 forms a central trimeric coiled‐coil structure upon activation, exposing hydrophobic grooves that serve as binding sites for HR2 (Gonepudi et al. 2026; Xu et al. 2004). The interaction between HR1 and HR2 culminates in the formation of a six‐helix bundle (6‐HB), effectively bringing viral and cellular membranes into proximity to facilitate fusion (L. Lu et al. 2014; C. Wang et al. 2018). Disrupting the HR1–HR2 interaction has been demonstrated as a viable antiviral strategy, as HR2‐derived peptides can competitively bind to HR1, preventing native 6‐HB formation and viral entry (Lu Lu et al. 2021; S. Xia et al. 2019). Similar strategies have been explored for MERS‐CoV, drawing parallels from HIV‐1 research, where HR2‐derived peptides like enfuvirtide (T20) have been successfully employed as fusion inhibitors (S. Xia et al. 2014). Peptides derived from the HR2 region can competitively bind to HR1, preventing the native HR1‐HR2 interaction and subsequent 6‐HB formation (S. Xia et al. 2019). The HR2‐derived peptide HR2P has demonstrated potent inhibitory effects on MERS‐CoV S‐mediated cell‐cell fusion and viral replication *in vitro* (Du et al. 2017). Modifications to HR2P, such as the introduction of hydrophilic residues, have further enhanced its stability, solubility, and antiviral activity, leading to the development of analogs like HR2P‐M2 (L. Lu et al. 2014). In addition to HR2P and its analogs, other HR2‐derived peptides have been identified with significant anti‐MERS‐CoV activity (Safiriyu et al. 2025). For instance, peptides derived from the HR2 region of bat coronavirus HKU4, such as HKU4‐HR2P2 and HKU4‐HR2P3, have shown potent inhibitory effects on MERS‐CoV S‐mediated cell‐cell fusion and pseudovirus infection, with IC₅₀ values in the submicromolar range(S. Xia et al. 2019). These studies provide strong experimental evidence that targeting the HR1–HR2 interaction is a validated and effective approach for coronavirus fusion inhibition.

The advancement of computational tools has revolutionized the drug discovery process, enabling *in silico* methods to predict and optimize peptide‐protein interactions (Alotaiq and Dermawan 2024; Alotaiq et al. 2024; Dermawan and Alotaiq 2025). Molecular docking and dynamics simulations provide insights into peptide candidates' binding affinities and conformational stability, facilitating the rational design of potent inhibitors (Alotaiq and Dermawan 2025b; Doni Dermawan et al. 2024). Such approaches have been instrumental in identifying HR2‐derived peptides with enhanced binding properties and antiviral efficacy. Despite these promising developments, challenges remain in translating HR2‐targeting peptides into clinical therapeutics. Peptide stability, bioavailability, and potential immunogenicity necessitate further optimization (Rosson et al. 2025). Strategies including peptide stapling, incorporating non‐natural amino acids, and conjugating with delivery systems are being explored to overcome these limitations and improve the pharmacokinetic profiles of peptide‐based antivirals (Ricardo et al. 2020; Xiao et al. 2025).

In this study, we aimed to identify and characterize antiviral peptides targeting the HR2 domain of the MERS‐CoV spike protein. Our primary objective was to evaluate the potential of these peptides to disrupt the critical HR1–HR2 interface essential for viral fusion. To achieve this, we employed computational approaches to examine peptide binding and stability and assessed their physicochemical properties and safety profiles. This study provides insights into the structural and biophysical features that govern peptide efficacy, offering a foundation for the rational design of potent and safe MERS‐CoV fusion inhibitors.

## 2 Materials and Methods

### 2.1 Selection and Preparation of Antiviral Peptides (AVPs)

To assemble a dataset of biologically relevant AVPs targeting the HR2 domain of the MERS‐CoV spike protein, we utilized the Antimicrobial Peptide Database (APD3) (Wang et al. 2016), a widely recognized repository of antimicrobial and antiviral peptides curated by the University of Nebraska Medical Center, Omaha, Nebraska, USA (last updated in January 2025). The data extracted included the APD ID, peptide name, amino acid sequence, and corresponding SwissProt and PDB IDs (where available). For peptides with experimentally determined structures (PDB ID), only structures with resolution ≤ 2.5 Å and complete backbone coordinates were included. Missing residues were modeled using MODELLER v10.3 (Šali and Blundell 1993), applying 100 optimization cycles and selecting the model with the lowest discrete optimized protein energy (DOPE) score. For peptides with only SwissProt entries and no known 3D structures, predictions were generated using AlphaFold3 (Jumper et al. 2021), and only models with an average predicted Local Distance Difference Test (pLDDT) score ≥ 45 were retained, ensuring high‐confidence structural predictions. Peptides lacking both PDB and SwissProt IDs or with pLDDT < 45 were excluded from further analysis. Peptides lacking both a PDB and SwissProt ID were excluded from further analysis due to insufficient structural data. The molecular size of each AVP (in kilodaltons, kDa) was calculated using the Protein Molecular Weight Calculator provided by the Science Gateway portal (Science‐Gateway 2025), based on the primary amino acid sequence. Additionally, binding site predictions were performed using Computed Atlas of Surface Topography of the universe of protein Folds (CASTpFold) (Ye et al. 2024) with a minimum probe radius of 1.4 Å and a threshold of ≥ 3 residues per pocket, identifying potential peptide‐target interaction sites. The number of predicted binding residues per peptide was recorded and used for prioritization.

As a reference for comparative docking and dynamic simulation analyses, Peptide‐6 (sequence: SLTQINWTLLDLTYEMESLQQVVKALNEYYIDLKHL) was employed as a standard HR2 inhibitor, based on previously validated *in vitro* findings (Kandeel et al. 2021). In a prior study, eight HR2‐derived peptides exhibited strong inhibition of spike‐mediated MERS‐CoV cell–cell fusion, with IC₅₀ values ranging from 0.25 to 2.3 µM. Peptide 4 (SLTQINWTLLDLTYEMESLQQVVKALNESYIDLKEL), peptide 5 (SLTQINWTLLDLTYEMESLQQVVKALNEYYIDLKEL), and peptide 6 (SLTQINWTLLDLTYEMESLQQVVKALNEYYIDLKHL) demonstrated particularly high efficacy, inhibiting 95%–98.3% of MERS‐CoV plaque formation. Specifically, Peptide‐4 achieved an EC₅₀ of 0.302 µM, confirming its potent antiviral activity. Importantly, none of the peptides exhibited cytotoxic effects at concentrations up to 10 µM, indicating a favorable safety profile (Kandeel et al. 2021). These findings support the therapeutic potential of HR2‐derived peptides and validate Peptide‐6 as a positive control in the context of this study. A compiled dataset containing peptide names, APD IDs, SwissProt or PDB IDs, molecular sizes, AlphaFold pLDDT scores (when applicable), amino acid sequences, and CASTpFold‐predicted binding residues is provided in Supplementary Data 1.

### 2.2 Peptide‐Protein Docking Simulation

Peptide‐protein docking simulations were carried out to elucidate the molecular interactions between selected AVPs and the HR2 domain of the MERS‐CoV spike protein. The primary objective of this analysis was to characterize the binding mechanisms of AVPs, including identification of key interacting residues, the nature of intermolecular forces, binding orientations, and overall binding affinities within the AVP–HR2 complexes. Prior to docking, the structural features and potential binding sites of the HR2 domain were analyzed using PDBsum (Laskowski et al. 2018), a specialized tool for generating comprehensive structural annotations and interaction summaries of protein complexes. This step allowed for a detailed mapping of the functional residues and spatial configuration of the HR2 domain involved in peptide recognition and binding (residue numbers: 1031, 1034, 1035, 1037, 1038, 1255, 1259, 1261, 1263, 1264, 1267, 1271). To ensure high‐quality input for docking simulations, the HR2 receptor structure was energy‐minimized and refined using Swiss‐PdbViewer v4.1.1 (Johansson et al. 2012), applying 500 steps of steepest descent and 1000 steps of conjugate gradient minimization, with GROMOS96 43B1 force field, and side‐chain optimization to correct minor structural inconsistencies and optimize side‐chain conformations. Following structural refinement, docking simulations were performed using the standalone version of HADDOCK v2.4 (High Ambiguity Driven Protein–Protein Docking) (Dominguez et al. 2003; van Zundert et al. 2016), with active residues defined at the HR2 interface (1031, 1034, 1035, 1037, 1038, 1255, 1259, 1261, 1263, 1264, 1267, 1271) and passive residues within 6.5 Å of the active residues; rigid‐body docking generated 1000 structures, semi‐flexible refinement involved 200 structures, and final water refinement produced 200 structures per peptide; clustering was based on RMSD cutoff of 2.0 Å, and top clusters were ranked by HADDOCK score (weighted sum: van der Waals 1.0, electrostatic 0.2, desolvation 1.0, AIR energy 0.1).

Each docking simulation generated a range of possible complex conformations, which were then evaluated based on two main criteria: the number of conformations per cluster (indicative of structural convergence and reliability) and the HADDOCK score, a weighted sum of energy terms that reflects the overall binding strength between the peptide and the protein. The top docked complexes per peptide were selected for further analysis, based on cluster size, HADDOCK score, and PRODIGY‐predicted binding free energy (ΔG ≥ 10.0 kcal/mol), ensuring high‐confidence and strongly bound complexes. To complement the docking evaluation, binding free energy predictions were conducted using PROtein binDIng enerGY prediction (PRODIGY) (Vangone and Bonvin 2017), with temperature set at 310 K and default threshold values for interfacial contacts; ΔG (kcal/mol) and dissociation constant (K_d) were calculated based on residue pairings, desolvation, and contact type. PRODIGY's calculations are based on the structural interface features of the docked models, including the number and type of interfacial contacts, desolvation energy, and residue pairing patterns. Together, HADDOCK and PRODIGY provided a comprehensive understanding of the structural and energetic landscape governing AVP–HR2 interactions, enabling the identification of peptides with high binding potential and favorable interaction profiles.

### 2.3 Molecular Dynamics (MD) Simulation

MD simulations were performed to explore the dynamic behavior and structural stability of AVP complexes with the HR2 domain of the MERS‐CoV spike protein. These simulations were carried out using GROMACS v2025.1, a widely adopted and highly efficient software suite for simulating the motions of biomolecular systems with atomic‐level detail (Pronk et al. 2013). The OPLS‐AA/L force field (Optimized Potentials for Liquid Simulations—All Atom/Long‐range) (Robertson et al. 2015) was employed to accurately model atomic interactions and force parameters within the peptide–protein complexes. A cubic simulation box with a 1.0 nm buffer around the complex was created to avoid edge effects and periodic boundary interactions. The system was solvated with SPCE (Single Point Charge Extended) (Yuet and Blankschtein 2010), and counterions were added to neutralize the system charge. Prior to production runs, energy minimization was performed using the steepest descent algorithm with a convergence criterion of 1000 kJ/mol/nm to remove steric clashes. A two‐step equilibration was conducted: first, an NVT ensemble for 500 ps at 310 K using the velocity‐rescale thermostat with a coupling constant of 0.1 ps, followed by an NPT ensemble for 500 ps at 1 bar using the Parrinello‐Rahman barostat with a coupling constant of 2 ps and isotropic pressure scaling. Periodic boundary conditions were applied in all directions, and long‐range electrostatics were treated with the Particle Mesh Ewald (PME) method with a cutoff of 1.2 nm. Van der Waals interactions were truncated at 1.2 nm with a force‐switching function applied from 1.0 to 1.2 nm (Alotaiq and Dermawan 2025a, 2025b).

Upon completion of equilibration, 100‐ns production MD simulations were run with a time step of 2 fs under NPT conditions at 310 K and 1 bar. Throughout the simulation period, various structural and energetic parameters were tracked to assess the integrity and performance of the complexes. These included RMSD to evaluate conformational stability, RMSF to measure residue‐level flexibility, RoG to gauge compactness, potential energy profiles, and the number of intermolecular hydrogen bonds, which are critical for peptide–protein interactions. Post‐simulation analyses were performed to visualize and interpret binding interactions and structural transitions. PyMOL v3.1.4 (Schrödinger 2020) and UCSF ChimeraX v1.9 (Pettersen et al. 2004) were employed for high‐resolution 3D visualization of the trajectories, enabling detailed inspection of binding residues, conformational changes, and interaction networks within the AVP–HR2 complexes. These visual and quantitative analyses provided comprehensive insights into the mechanistic aspects of antiviral peptide binding, supporting the rational assessment of candidate peptides as potential fusion inhibitors targeting the MERS‐CoV HR2 domain.

#### 2.3.1 Molecular Mechanics/Poisson–Boltzmann Surface Area (MM/PBSA) Calculations

To further elucidate the energetics underlying the interaction between AVPs and the HR2 domain of the MERS‐CoV spike protein, Molecular Mechanics/Poisson–Boltzmann Surface Area (MM/PBSA) calculations were conducted. This computational method combines molecular mechanics energy terms with solvation effects to estimate the binding free energy of biomolecular complexes (Tian et al. 2014). Following the 100 ns MD simulations, 2,000 representative conformational snapshots were extracted from the last 20 ns of each trajectory at 10 ps intervals to ensure equilibrium‐state sampling and statistical robustness. These frames were selected to provide a statistically robust sampling of the conformational space occupied by the complex during the simulation. Calculations were carried out using the gmx_MMPBSA tool (Miller et al. 2012; Valdés‐Tresanco et al. 2021), an integrated script compatible with the GROMACS platform, that facilitates the efficient computation of binding energies across MD‐generated ensembles. This tool incorporates standard MM/PBSA workflows and supports precise evaluation of interaction thermodynamics for biomolecular complexes (Panday and Alexov 2022). For each snapshot, MM/PBSA calculations were performed to decompose the total binding energy into multiple energetic components, including van der Waals energy (ΔE_vdW), electrostatic energy (ΔE_elec), polar solvation energy (ΔG_polar), and non‐polar solvation energy (ΔG_nonpolar). The polar solvation term was calculated using the Poisson–Boltzmann model with a solute dielectric constant of 2, solvent dielectric constant of 80, ionic strength of 0.15 M, and a grid spacing of 0.16 nm. The non‐polar solvation energy was estimated using the solvent‐accessible surface area (SASA) model with a surface tension constant of 0.022 kJ/mol/Å² and a probe radius of 1.4 Å. Entropic contributions (− TΔS) were not included due to their high computational cost and limited impact on comparative ranking. The binding free energy (ΔG_binding) of each complex was calculated using the following thermodynamic expression:

ΔG_binding=ΔG_complex−ΔG_peptideX−ΔG_proteinY,

where

ΔG_binding: the binding free energy associated with forming the peptide‐protein complex.

ΔG_complex: the free energy of the fully solvated peptide‐protein complex.

ΔG_peptideX: the free energy of AVP in its solvated state when unbound.

ΔG_proteinY: the free energy of HR2 in its solvated state when unbound.

This approach allowed for the quantification of energetic changes upon peptide binding, offering valuable insights into interaction strength, stability, and the likelihood of effective inhibition of the HR2 fusion interface. The MM/PBSA method was critical in identifying peptides with the most favorable thermodynamic profiles for disrupting MERS‐CoV membrane fusion by integrating structural and energetic analyses.

### 2.4 Haemolytic AVPs

To assess the potential cytotoxic effects of AVPs, particularly their propensity to induce red blood cell lysis, the hemolytic activity of the selected peptides was evaluated using HemoPI2 (Rathore et al. 2025), an advanced web‐based predictive tool. This platform applies machine learning algorithms trained on datasets comprising experimentally validated hemolytic and non‐hemolytic peptides to estimate the likelihood of hemolysis based on sequence features. All predictions were performed using the default server parameters with the Hybrid1 classification model selected, peptide length range set automatically by the server, and no sequence modifications applied. The Hybrid1 classification model within HemoPI2 was employed for this study due to its enhanced predictive performance. This model integrates two complementary techniques: ESM2‐t6, a transformer‐based protein language model that captures contextual information from peptide sequences, and MERCI (Motif‐EmeRging and with Classes‐Identification), which identifies motifs typically associated with hemolytic peptides. The hybrid model combines the strengths of both sequence‐wide and motif‐based analyses. A threshold value of 0.58 was used as the cut‐off for classification, in accordance with the model's validated parameters for balancing sensitivity and specificity. The prediction was performed in binary classification mode (hemolytic vs. non‐hemolytic), and a threshold value of 0.58 (default server cut‐off) was used for the hybrid score to balance sensitivity and specificity. Peptides with hybrid scores ≥ 0.58 were classified as hemolytic, whereas those with scores < 0.58 were classified as non‐hemolytic. Each AVP sequence, including the standard inhibitor (Peptide‐6), was processed through HemoPI2 to obtain three key outputs: the ESM2 score, reflecting contextual sequence confidence; the MERCI score, denoting motif‐driven associations; and the hybrid score, a composite index representing the combined predictive power of both methods. The final prediction output, categorized as either hemolytic or non‐hemolytic, was determined by whether the hybrid score exceeded the defined threshold. A hybrid score approaching 1.00 indicated a high probability of hemolytic activity, suggesting potential safety concerns, whereas a score nearing 0.00 implied non‐hemolytic behavior and improved biocompatibility. The integration of hemolytic activity predictions helped ensure that the selected candidates maintained a desirable balance between antiviral efficacy and minimal host toxicity.

### 2.5 Physicochemical Characterization of AVPs

The physicochemical profiles of the selected AVPs, including the standard inhibitor (Peptide‐6), were systematically evaluated using the ProtParam tool (ExPASy Bioinformatics Resource Portal) (Gasteiger et al. 2005). This tool computes a range of biochemical and biophysical parameters directly from amino acid sequences, enabling *in silico* assessment of peptide drug‐likeness and stability. For each AVP, we determined the theoretical isoelectric point, extinction coefficient (expressed in M⁻¹ cm⁻¹ at 280 nm), estimated half‐life in mammalian reticulocytes (*in vitro*), instability index, aliphatic index, and GRAVY. The extinction coefficient was calculated based on the number of tyrosine, tryptophan, and cystine residues, which contribute to UV absorbance at 280 nm. The estimated half‐life was predicted using the N‐end rule, reflecting peptide stability in mammalian cells. The instability index was used to classify peptides as either stable (≤ 40.00) or unstable (> 40.00), indicating their susceptibility to degradation. The aliphatic index, which reflects the relative volume occupied by aliphatic side chains (alanine, valine, isoleucine, and leucine), served as an indicator of thermostability. Meanwhile, GRAVY values were computed by averaging the hydropathy values of all amino acids in the peptide, offering insights into overall hydrophilicity or hydrophobicity. Together, these physicochemical descriptors provide valuable insights into each AVP's solubility, stability, and potential bioavailability, thereby informing the selection of promising candidates for further structural and functional validation.

## 3 Results

### 3.1 Selection and Structural Profiling of AVPs

Out of 264 AVPs initially obtained from the APD3, a refined set of 161 peptides with structural identifiers (SwissProt and/or PDB ID) was prioritized for structure‐based computational analysis. This selection ensured sufficient structural fidelity for downstream modeling, interaction prediction, and simulation studies. Table 1 presents a representative sample of 10 randomly selected AVPs from the dataset to illustrate the diversity in molecular and structural characteristics across the selection. Peptides range in molecular size from 2.32 to 12.69 kDa, reflecting a broad spectrum of sequence lengths and structural complexities. For example, Griffithsin, the largest peptide in this subset, spans 12.69 kDa and possesses multiple predicted binding residues (e.g., positions 4, 6, 12, 16–18, and 56–58), indicating extensive potential for multivalent interactions. In contrast, smaller peptides like Brevinin‐2 and Varv peptide E (2.32 and 2.92 kDa, respectively) have more localized binding sites but may benefit from compact structures that facilitate target accessibility.

**Table 1:** Structural and database annotations of selected antiviral peptides (AVPs), including size, confidence scores, and predicted binding sites. This table presents a representative subset of 10 randomly selected AVPs.

Anviral Peptide	APD ID	SwissProt ID/PDB ID	Size (kDa)	Average pLDDT	Peptide Binding Sites (Position of Residues)
An1a	AP03266	A0A5Q1NCA8	6.93	83.62	20, 21, 23, 24, 25, 27, 28, 30, 55
Brevinin‐2	AP00599	P32424	2.32	81.49	1, 2, 12, 13, 21, 22
CCL20	AP02075	1M8A	7.96	N/A	5, 9, 11, 14, 15, 16, 17, 20, 25, 29, 37, 40, 46, 48, 51, 55, 63
Griffithsin	AP02133	2GTY	12.69	N/A	4, 6, 12, 16, 17, 18, 26, 27, 28, 35, 56, 57, 58
Human defensin 5	AP00180	1ZMP	3.59	N/A	3, 6, 7, 8, 9, 15, 31
Lactoferricin B	AP00026	1LFC	3.13	N/A	11, 12, 13, 15
Neutrophil cationic peptide 1 type B	AP00174	Q64365	3.84	63.00	1, 2, 3, 6, 7, 13, 14, 17, 18
Piscidin 2	AP01649	Q8UUG2	9.11	74.76	14, 20, 23
Shepherin II	AP00512	Q9FR52	3.26	81.04	7, 9
Varv peptide E	AP01030	P83835	2.92	90.55	20, 27, 28, 29

*Note:* The complete list of 161 AVPs and detailed annotations is available in Supplementary Data 1.

The predicted binding site residues vary significantly among peptides. Highly structured peptides such as CCL20 and Human defensin five display densely distributed binding hotspots across the sequence, suggesting potential for extensive interface complementarity with protein receptors. Notably, An1a shows concentrated binding regions clustered in the C‐terminal half of the peptide (residues 20–30 and 55), indicating a potentially focused interaction motif. Structural confidence, assessed through AlphaFold3 average pLDDT scores, was available for seven of the ten peptides in this subset. The scores ranged from moderate (e.g., 63.00 for Neutrophil cationic peptide 1 type B) to very high (e.g., 90.55 for Varv peptide E), underscoring variation in model reliability. Peptides with high pLDDT values (> 80) generally exhibited well‐folded domains suitable for detailed binding energy calculations. Peptides with existing crystal or NMR structures (e.g., Griffithsin, CCL20, Lactoferricin B) were not assigned pLDDT values, as their tertiary structures were experimentally determined.

#### 3.1.1 Molecular Docking of AVPs to the HR2 Target Site

**Figure 1:**
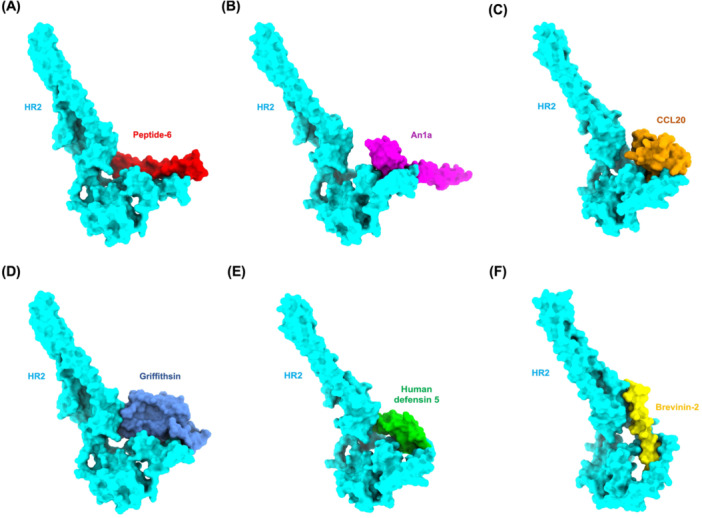
Structural visualization of docked complexes between the HR2 domain of the MERS‐CoV spike protein and top‐ranked antiviral peptides (AVPs). Surface‐rendered models illustrate the binding interactions between the HR2 domain of the MERS‐CoV spike protein (shown in cyan) and selected AVPs, each represented in a distinct color. (A) Peptide‐6 (red) is a reference inhibitor that binds peripherally along the HR2 surface. (B) An1a (magenta) penetrates deeper into the HR2 groove, suggesting robust interference with the fusion core formation. (C) CCL20 (orange) exhibits compact and stable surface binding at the HR2 site. (D) Griffithsin (slate blue) engages a broad contact interface, which is consistent with its low HADDOCK score. (E) Human Defensin 5 (green) interacts with a moderate surface area, reflecting intermediate docking performance. (F) Brevinin‐2 (yellow) binds deeply within a hydrophobic cleft of HR2, indicating high structural complementarity and potential inhibitory efficiency.

The HR2 domain is a crucial target in disrupting viral entry by interfering with 6‐HB formation, a conserved mechanism in class I viral fusion proteins. We employed HADDOCK‐based docking, complemented with PRODIGY‐derived binding affinity (ΔG) estimations and KD (dissociation constant) predictions. A binding affinity threshold of −10.0 kcal/mol was set to identify promising AVP candidates, using Peptide‐6 (ΔG = −7.0 kcal/mol) as a standard inhibitor. Peptide‐6, shown in red, exhibits a relatively peripheral binding mode along the surface of the HR2 domain (cyan) (Figure 1A). While it aligns along a shallow groove, the interaction appears more surface‐level and linear, suggesting moderate blocking potential. This configuration may limit its ability to effectively interfere with the structural rearrangements required for viral membrane fusion. The docking results, summarized in Table 2, revealed that all selected AVPs surpassed the binding affinity threshold of −10.0 kcal/mol, suggesting a strong and energetically favorable interaction landscape with the HR2 domain. This consistent binding efficacy across diverse AVP structures reinforces the conserved nature of the HR2 pocket and its potential as a viable therapeutic target in disrupting viral fusion mechanisms. Among the tested AVPs, An1a recorded the highest binding affinity (ΔG = −11.5 kcal/mol) and a substantial HADDOCK score of −69.0 a.u., with a cluster size of 19 and a root mean square deviation (RMSD) of 1.6 Å. These parameters indicate a highly stable and reproducible docking conformation. An1a, visualized in magenta (Figure 1B), adopts a deep‐penetrating binding pose, anchoring itself well into the hydrophobic cleft of HR2. This orientation suggests stronger steric hindrance against the 6‐HB formation, a critical step in class I viral fusion. The peptide's extended surface contact implies high‐affinity binding and potentially broad‐spectrum inhibitory activity, especially given its excellent docking parameters. Melittin, a well‐studied membrane‐active peptide, was closely followed with a binding affinity of −11.2 kcal/mol, a HADDOCK score of −65.7 a.u., and an RMSD of 1.9 Å. Despite its known cytolytic activity, the docking results suggest Melittin's amphipathic helical structure aligns effectively within the HR2 interface, facilitating energetically stable binding.

**Table 2:** Molecular docking and binding affinity parameters of antiviral peptides (AVPs) targeting the HR2 domain of MERS‐CoV spike protein.

AVP‐HR2 Complex	HADDOCK Score (a.u.)	Binding Affinity ΔG (kcal/mol)	Kd (M)	Cluster size	RMSD (Å)
HR2_Peptide‐6 (standard inhibitor)	−45.5 ± 4.6	−7.0	1.10E‐05	6	1.8 ± 0.3
HR2_An1a	−69.0 ± 10.8	−11.5	8.00E‐09	19	1.6 ± 0.3
HR2_Melittin	−65.7 ± 11.0	−11.2	1.20E‐08	17	1.9 ± 1.2
HR2_CCL20	−77.7 ± 12.2	−10.9	1.90E‐08	10	1.4 ± 0.2
HR2_Labyrinthopeptin A2	−80.3 ± 16.7	−10.9	2.20E‐08	9	1.1 ± 0.1
HR2_Lactoferricin B	−64.1 ± 11.3	−10.8	2.40E‐08	8	1.1 ± 0.1
HR2_Griffithsin	−84.4 ± 12.2	−10.7	2.80E‐08	16	1.7 ± 0.9
HR2_Shepherin II	−43.7 ± 3.7	−10.6	3.50E‐08	9	1.7 ± 0.3
HR2_Human neutrophil peptide‐1	−42.4 ± 10.6	−10.5	3.70E‐08	8	1.0 ± 0.7
HR2_Myticin C	−64.2 ± 5.1	−10.5	4.00E‐08	27	1.6 ± 0.1
HR2_Tricyclic peptide RP 71955	−66.7 ± 5.6	−10.5	3.70E‐08	6	1.4 ± 0.1
HR2_Human defensin 5	−52.3 ± 18.5	−10.4	4.60E‐08	5	0.9 ± 0.6
HR2_Human defensin hBD‐1	−56.1 ± 34.7	−10.3	5.40E‐08	5	1.4 ± 0.9
HR2_Neutrophil cationic peptide 1 type B	−39.0 ± 16.4	−10.3	5.80E‐08	5	1.7 ± 0.3
HR2_Brevinin‐2	−70.8 ± 8.9	−10.2	6.20E‐08	5	1.5 ± 0.5
HR2_Latarcin 1	−56.5 ± 16.3	−10.2	6.50E‐08	8	2.0 ± 0.1
HR2_Human neutrophil peptide‐2	−70.3 ± 9.4	−10.1	7.70E‐08	6	1.6 ± 0.2
HR2_Piscidin 2	−86.9 ± 15.8	−10.0	9.40E‐08	5	1.6 ± 1.6
HR2_Varv peptide E	−42.8 ± 10.1	−10.0	8.60E‐08	7	1.1 ± 0.6

Meanwhile, CCL20, an endogenous chemokine with known antimicrobial activity, achieved a ΔG of −10.9 kcal/mol and a HADDOCK score of −77.7. The structural model (Figure 1C) reveals that CCL20 interacts compactly with the HR2 region, utilizing a broad surface area to form multiple stabilizing contacts, as evidenced by its low RMSD of 1.4 Å. Similarly, Labyrinthopeptin A2, a lantibiotic with complex ring topology, exhibited a notable HADDOCK score of −80.3 a.u. and ΔG of −10.9 kcal/mol, supported by an RMSD of 1.1 Å, suggesting a tightly packed and highly stable interaction. Griffithsin, a mannose‐binding lectin with broad‐spectrum antiviral properties, demonstrated the most favorable HADDOCK score at −84.4 a.u., indicative of an exceptionally stable complex with HR2. Despite a slightly lower binding affinity ( −10.7 kcal/mol), its large cluster size (*n* = 16) and a low RMSD of 1.7 Å indicate that the docking simulation converged consistently to a well‐defined binding pose (Figure 1D). The peptide spans a large area of the HR2 interface, likely stabilizing the complex via multiple hydrogen bonds and polar contacts. Its spatial occupation of shallow and moderately recessed grooves reinforces its antiviral potential, albeit limited by pharmacokinetic liabilities like a shorter half‐life.

Human Defensin 5 (HD5) binds to a shallow pocket on the HR2 surface. Although it retains a respectable binding energy (ΔG = −10.4 kcal/mol), its smaller interaction surface area and cluster size suggest less conformational adaptability (Figure 1E). As a result, HD5 may offer only partial blockade of HR2 activity, potentially functioning more effectively in synergy with other peptides or inhibitors. Brevinin‐2, although presenting a modest binding affinity of −10.2 kcal/mol compared to the top candidates, struck an optimal balance among docking parameters: a solid HADDOCK score (− 70.8 a.u.), favorable RMSD (1.5 Å), and a compact binding orientation as shown in Figure 1F. The docking visualization reveals that Brevinin‐2 accesses the deeper region of the HR2 binding pocket, allowing for extensive hydrophobic and hydrogen bonding interactions, thereby ensuring strong anchoring and potential to block HR2‐mediated viral fusion events. In contrast, while Griffithsin and Labyrinthopeptin A2 demonstrate superior docking energetics, their structural complexity and potentially lower stability *in vivo* might pose formulation challenges. Therefore, although binding affinity and HADDOCK scores are critical for identifying potent inhibitors, complementary parameters such as structural convergence (RMSD), reproducibility (cluster size), and predicted pharmacokinetics are essential for prioritizing AVPs for further experimental validation. The complete molecular docking results can be seen in Supplementary Data 2.

The atomic contact analysis between AVPs and the HR2 domain of the MERS‐CoV spike protein revealed intricate interaction patterns across a variety of physicochemical contact types. These interactions, broken down into charged–charged, charged–polar, charged–apolar, polar–polar, polar–apolar, and apolar–apolar atomic contacts, provide deeper insights into the binding mechanisms underlying peptide‐HR2 complex stability. The number of each contact type, along with the non‐interacting surface (NIS) area categorized by charge polarity, gives a comprehensive structural profile of each AVP–HR2 interaction interface (Table 3). High‐affinity peptides such as An1a, Melittin, and Griffithsin exhibited extensive interactions across polar and apolar categories. Notably, An1a and Melittin formed a large number of charged–apolar (17 and 12, respectively) and polar–apolar (20 and 24, respectively) contacts, which are critical for stabilizing peptide orientation in the amphipathic groove of the HR2 domain. This abundance of mixed hydrophilic and hydrophobic contacts may contribute to their exceptional binding energies reported in earlier docking analyses. Griffithsin, the peptide with the strongest HADDOCK score, displayed the highest number of charged–polar (11) and charged–apolar (25) contacts, along with substantial apolar–apolar contacts (23), supporting its robust and multi‐faceted binding interface.

**Table 3:** Intermolecular contacts and non‐interacting surface areas of AVP‐HR2 complexes. This table summarizes the types and frequencies of atomic interactions between antiviral peptides and the HR2 domain, highlighting charged, polar, and apolar contacts alongside their respective non‐interacting surface areas.

AVP‐HR2 complex	ICs charged‐charged	ICs charged‐polar	ICs charged‐apolar	ICs polar‐polar	ICs polar‐apolar	ICs apolar‐apolar	NIS charged	NIS apolar
HR2_Peptide‐6 (standard inhibitor)	2	5	3	2	7	3	16.13	44.84
HR2_An1a	3	3	17	1	20	21	15.20	46.50
HR2_Melittin	3	4	12	4	24	21	16.11	46.31
HR2_CCL20	4	5	19	3	18	20	17.48	44.79
HR2_Labyrinthopeptin A2	2	9	19	8	22	12	16.39	44.59
HR2_Lactoferricin B	3	8	13	0	18	16	17.06	45.15
HR2_Griffithsin	3	11	25	7	17	23	15.90	44.47
HR2_Shepherin II	1	6	13	1	19	21	13.78	48.08
HR2_Human neutrophil peptide‐1	0	6	11	2	20	20	16.05	45.15
HR2_Myticin C	1	5	6	7	27	17	15.45	46.88
HR2_Tricyclic peptide RP 71955	0	0	12	3	21	26	14.78	46.74
HR2_Human defensin 5	3	4	11	2	18	16	16.78	44.41
HR2_Human defensin hBD‐1	3	10	10	4	20	21	16.07	45.25
HR2_Neutrophil cationic peptide 1 type B	1	4	8	3	22	20	17.76	45.63
HR2_Brevinin‐2	0	2	12	1	18	31	15.74	46.23
HR2_Latarcin 1	5	9	25	2	11	12	18.46	44.30
HR2_Human neutrophil peptide‐2	1	5	10	1	17	28	15.72	45.15
HR2_Piscidin 2	2	6	17	0	13	22	17.00	45.33
HR2_Varv peptide E	1	2	2	6	25	19	15.15	46.13

Abbreviations: ICs, Number of intermolecular contacts; NIS, Non‐interacting surface.

Peptides such as Labyrinthopeptin A2 and CCL20 also exhibited diverse contact profiles. Labyrinthopeptin A2 showed a relatively balanced spread of interactions, with significant numbers of polar–polar (8) and charged–polar (9) contacts, indicating that hydrogen bonding and electrostatic complementarity play a strong role in its binding stability. Its NIS values (16.39% charged, 44.59% apolar) were in line with a relatively well‐encapsulated interface, suggesting efficient utilization of binding surface area. Similarly, CCL20 exhibited a high number of charged–apolar (19) and apolar–apolar (20) contacts, suggesting deep embedding into the hydrophobic core of HR2, complemented by a charged NIS of 17.48%, the highest among top binders, possibly contributing to its notable interface complementarity. Interestingly, Brevinin‐2, despite a slightly lower binding affinity (−10.2 kcal/mol), exhibited a remarkably high number of apolar–apolar contacts (31) (the highest among all peptides), highlighting strong van der Waals interactions that may contribute to its favorable structural stability and pharmacokinetics. It had one of the lowest polar contact counts, underscoring its reliance on hydrophobic packing rather than polar or ionic interactions. On the other hand, Peptide‐6, the standard inhibitor, demonstrated limited interaction versatility, with only 2 charged–charged and 3 charged–apolar contacts. Its relatively low binding affinity (−7.0 kcal/mol) and modest NIS values (16.13% charged, 44.84% apolar) indicate weaker and less specific interactions, aligning with its lower performance in docking simulations. Several other peptides, such as Tricyclic peptide RP 71955 and Myticin C, also showed strong hydrophobic contributions, with high apolar–apolar contact numbers (26 and 17, respectively) and relatively large polar–apolar interfaces. These features are consistent with stable docking conformations in their cluster RMSDs and suggest favorable entropic contributions during binding. The complete molecular interactions are provided in Supplementary Data 3.

**Figure 2:**
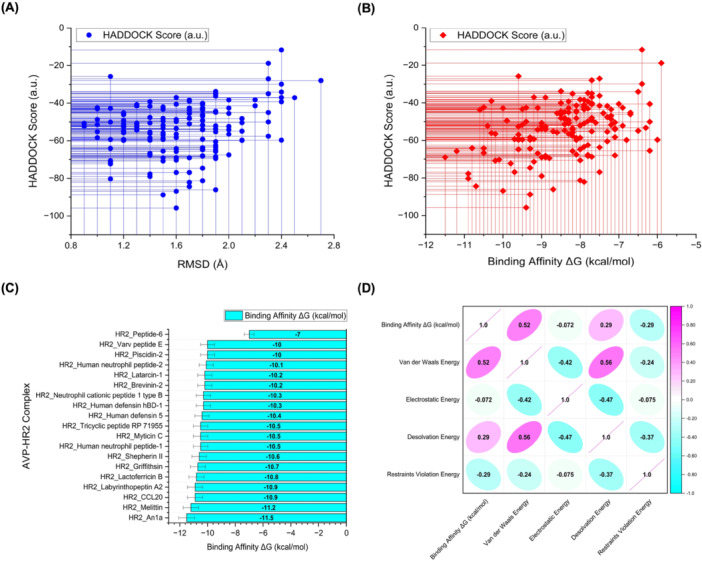
Molecular docking analysis of antiviral peptides (AVPs) targeting the HR2 domain. (A) Scatter plot showing the correlation between HADDOCK scores (a.u.) and root‐mean‐square deviation (RMSD, Å) across all docked AVP–HR2 complexes. Lower HADDOCK scores with low RMSD values indicate more stable and structurally consistent binding conformations. (B) Scatter plot illustrating the relationship between HADDOCK scores and predicted binding affinities (ΔG, kcal/mol). A general trend is observed where more negative HADDOCK scores correlate with stronger binding affinities. (C) Bar graph ranking the top‐performing AVP–HR2 complexes based on their predicted binding affinity (ΔG), with a cutoff value of −10.0 kcal/mol. (D) Correlation matrix depicting the relationships between binding affinity and individual energetic components, including van der Waals, electrostatic, desolvation, and restraint violation energies. Correlation coefficients range from −1 to +1, with values closer to ±1 indicating strong linear relationships.

Figure 2A presents a scatter plot of the HADDOCK scores (in arbitrary units) plotted against the RMSD (Å) for each docking pose. The HADDOCK score represents a weighted sum of several energy terms, including van der Waals, electrostatic, desolvation, and restraint energies. In general, lower HADDOCK scores denote more favorable and stable docking interactions. A visual inspection of the plot shows a dense clustering of data points in the RMSD range between 1.2 and 1.8 Å, which indicates that the docked complexes share a common binding orientation or convergence pattern around a preferred pose. Moreover, this distribution suggests that many peptide candidates are capable of binding HR2 with consistent and reproducible geometries. The HADDOCK scores range from around −20 to as low as −100, with the most favorable (lowest) scores associated with RMSD values closer to 1.2 Å. This supports the reliability of the docking results, implying that multiple docking simulations yielded similar conformations, reinforcing the structural stability of the peptide‐HR2 complexes. Overall, this panel highlights that favorable binding interactions are not outliers but a repeated trend across several peptides. Figure 2B further investigates the energetic landscape by plotting HADDOCK scores against the predicted binding affinity (ΔG, kcal/mol) for each complex. As expected, a general inverse relationship is observed: more negative HADDOCK scores (indicating favorable binding) are associated with more negative ΔG values, signifying stronger predicted binding affinities. This correlation validates the HADDOCK scoring function as being qualitatively consistent with thermodynamic predictions. However, the relationship is not strictly linear, and some dispersion is noticeable. This deviation may stem from differences in individual energy contributions, such as electrostatic versus van der Waals interactions, or desolvation effects that may influence ΔG differently than HADDOCK scoring weights. Importantly, a significant number of complexes cluster around a ΔG of −10 to −11 kcal/mol, coupled with HADDOCK scores between −60 and −100, indicating highly favorable interactions and robust complex formation potential.

Figure 2C provides a ranked bar chart summarizing the binding affinities (ΔG, kcal/mol) of 20 AVPs docked with HR2. This visualization identifies the top‐performing peptide candidates. HR2_Ana‐1 exhibits the most favorable binding affinity at −11.5 kcal/mol, followed closely by HR2_Melittin (− 11.2 kcal/mol) and HR2_CCL20 (− 11.0 kcal/mol). These values suggest strong molecular interactions, likely due to complementary shape, electrostatics, and hydrophobic interactions at the binding interface. Other peptides such as HR2_Labyrinthopeptin A2, HR2_Lactoferricin B, and HR2_Griffithsin also demonstrate potent binding with ΔG values ranging from −10.5 to −10.8 kcal/mol, suggesting their potential as therapeutic leads. This strong performance likely results from the amphipathic and cationic nature of AVPs, which allows them to interact with protein targets efficiently. Figure 2D displays a correlation matrix (numerical values and ellipses) for the various energetic contributions influencing binding affinity. The binding affinity (ΔG) shows a positive correlation with van der Waals energy (*r* = 0.52) and desolvation energy (*r* = 0.29), indicating these components are primary drivers of strong binding in the AVP–HR2 complexes. Notably, electrostatic energy has almost no correlation with ΔG (*r* = −0.072), suggesting that polar interactions or charged residues are less influential in this specific interaction model than hydrophobic or packing forces. The negative correlation between restraint violation energy and ΔG (*r* = −0.29) implies that binding affinity improves when fewer violations in docking restraints occur, supporting the validity of the top‐ranked models. This analysis suggests that hydrophobic interactions and optimal structural accommodation (i.e., low RMSD and low restraint violations) are the most crucial determinants for effective AVP binding to HR2.

Based on the provided molecular docking and interaction analysis results, a detailed examination of hydrogen bonding interactions between AVPs and the HR2 domain reveals a consistent binding pattern with the standard inhibitor, Peptide‐6 (Table 4). This observation is especially evident at key residues Glu1265 and Ser1268, which play crucial roles in stabilizing peptide interactions at the HR2 binding site. Peptide‐6, used as a reference standard due to its established inhibitory role, forms multiple hydrogen bonds with HR2 residues, including Glu1265 and Ser1268. Specifically, Glu1265 forms a hydrogen bond with Ser18 (OG) at a distance of 2.87 Å, while Ser1268 interacts with Lys24 (NZ) at a distance of 2.79 Å. These interactions are critical for anchoring the inhibitor within the HR2 pocket and enhancing binding affinity, which is reflected by its favorable HADDOCK score and binding energy profile. Several AVPs replicate these key hydrogen bonding patterns, indicating similar binding behavior and potential inhibitory function. The AVP Ana‐1a exhibits two hydrogen bond interactions involving Glu1265: one with Phe26 (N, 3.11 Å) and another with Gly27 (N, 2.67 Å). Additionally, Ana‐1a forms a hydrogen bond with Ser1268 via His38 (NE2, 2.99 Å). These interactions closely mirror those of Peptide‐6, suggesting that Ana‐1a may effectively mimic the binding mode of the standard inhibitor. Griffithsin, another potent AVP, also engages HR2 residue Glu1265 through a hydrogen bond with Gln102 (NE2, 2.68 Å), while Ser1268 forms a hydrogen bond with Arg5 (N, 2.90 Å). These interactions further underscore the AVP's ability to engage critical HR2 residues, reinforcing the hypothesis of similar binding mechanisms. Griffithsin also shows additional interactions with other HR2 residues, such as Tyr1264 and Leu1260, enhancing its binding stability. Brevinin‐2, likewise, forms hydrogen bonds with both Glu1265 and Ser1268. Specifically, Glu1265 (SD) interacts with Lys28 (NZ, 3.12 Å), while Ser1268 is involved in two separate hydrogen bonds with Ser5 (OG, 2.70 Å) and Leu2 (N, 3.17 Å). Although the distances vary slightly, preserving hydrogen bonding at these crucial residues highlights Brevinin‐2's potential for HR2 targeting.

**Table 4:** Key residue‐level interactions between HR2 and antiviral peptides (AVPs). The molecular docking simulation identified hydrogen bonds and polar contact distances between HR2 residues and interacting AVP residues.

AVP‐HR2 complex	Residue (receptor)	Protein atom (receptor)	Residue (interacting protein/peptide)	Protein atom (interacting protein/peptide)	Interaction distance (Å)
HR2_Peptide‐6 (standard inhibitor)	Thr1258	N	Glu15	OE2	2.98
	Thr1258	OG1	Glu15	OE2	2.74
	Leu1260	N	Tyr14	OH	2.96
	Asp1261	O	Ser18	OG	2.93
	Glu1265	OE2	Ser18	OG	2.87
	Ser1268	OG	Lys24	NZ	2.79
	Gln1271	NE2	Glu28	OE1	2.72
HR2_An1a	Asp1261	N	Leu21	O	3.21
	Glu1265	OE2	Phe26	N	3.11
	Glu1265	OE2	Gly27	N	2.67
	Ser1268	OG	His38	NE2	2.99
HR2_Griffithsin	Thr1253	OG1	Gly36	O	3.26
	Leu1260	N	Ser19	OG	2.85
	Tyr1264	OH	His4	ND1	2.85
	Tyr1264	OH	Glu119	OE2	3.21
	Glu1265	OE2	Gln102	NE2	2.68
	Ser1268	OG	Arg5	N	2.90
HR2_Brevinin‐2	Thr1258	O	Lys31	NZ	2.67
	Glu1265	SD	Lys28	NZ	3.12
	Ser1268	O	Ser5	OG	2.70
	Ser1268	OG1	Leu2	N	3.17

#### 3.1.2 Structural Dynamics and MM/PBSA‐Based Evaluation of AVP–HR2 Complexes

**Figure 3:**
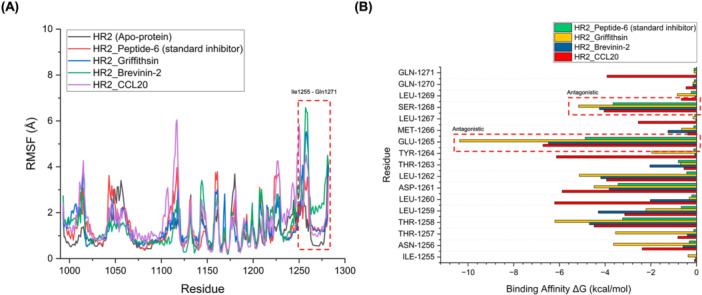
Comparative analysis of flexibility and binding contributions of AVPs on the HR2 domain. (A) Root Mean Square Fluctuation (RMSF) profiles of HR2 in apo form and complexes with selected AVPs, highlighting dynamic changes around residues Ile1255–Gln1271. (B) Per‐residue MM/GBSA binding free energy decomposition for top AVPs, pinpointing key interacting residues (Glu1265 and Ser1268) contributing to antagonistic activity.

To understand the biophysical basis underlying the inhibitory activity of selected AVPs against the HR2 domain of the MERS‐CoV spike protein, MD simulations were conducted for each AVP–HR2 complex. These simulations were benchmarked against Peptide‐6, a known HR2‐targeting standard inhibitor, to assess conformational stability, residue‐level flexibility, and interaction strength. Following docking (HADDOCK score and cluster size evaluation), binding free energy screening (|ΔG_binding | ≥ 10.0 kcal/mol), and safety profiling (hybrid hemolysis score < 0.58), three peptides—Griffithsin, Brevinin‐2, and CCL20—were advanced for detailed structural dynamics and MM/PBSA analyses as final candidate AVPs. These peptides demonstrated binding and stability metrics comparable to or exceeding those of the standard inhibitor Peptide‐6. The root mean square fluctuation (RMSF) profiles (Figure 3A) revealed the time‐averaged positional fluctuations of individual residues within the HR2 domain in both apo and peptide‐bound states. Across most of the HR2 backbone, the complexes demonstrated a conserved pattern of low fluctuation, indicating overall structural rigidity. However, a notable increase in residue flexibility was consistently observed in the Ile1255–Gln1271 region, an essential region involved in the fusion process that undergoes conformational rearrangement during the pre‐fusion to post‐fusion transition. This elevated fluctuation peak is particularly informative, as it reflects dynamic motions that could either facilitate or inhibit conformational changes depending on the nature of peptide binding. In the case of Peptide‐6, this region exhibited moderate but localized flexibility, consistent with partial stabilization that interferes with the necessary conformational dynamics for membrane fusion. Such behavior is characteristic of effective HR2 antagonists, which bind in a manner that disrupts critical intramolecular interactions (hydrogen bonds), thereby locking HR2 in a non‐functional state. Interestingly, Griffithsin (blue), Brevinin‐2 (green), and CCL20 (purple) demonstrated RMSF profiles that closely mirrored that of Peptide‐6 (red) in the Ile1255–Gln1271 region. This congruence in fluctuation amplitude and position suggests that these peptides likely engage the HR2 helix with similar binding mechanics, potentially destabilizing the local hydrogen‐bond network essential for the heptad repeat's structural rearrangement. Such disruption likely hinders the 6‐HB formation required for fusion, reinforcing their proposed antagonist‐like mode of action.

The MM/PBSA‐based per‐residue binding free energy decomposition (Figure 3B) provides insights into the key HR2 residues contributing to AVP affinity. Critical residues such as Glu1265 and Ser1268 exhibited highly favorable ΔG binding contributions across all top‐performing AVPs and Peptide‐6. Particularly, Glu1265, previously identified as part of the fusion antagonist hotspot, showed consistently substantial binding contributions across Peptide‐6, Griffithsin, Brevinin‐2, and CCL20. This suggests that these AVPs occupy the same antagonist binding pocket and interact with HR2 in a mechanistically conserved manner. Similarly, Ser1268 displayed comparable energy profiles, reinforcing the notion of convergent binding behavior among these peptides. Interestingly, while the peptides varied in their amino acid sequences and origins (Griffithsin, Brevinin‐2, and CCL20), they converged on similar biophysical interaction patterns with HR2. This is evident from the overlapping energy and the alignment of their high‐binding residues with those of Peptide‐6. The antagonistic region highlighted in red (residues Glu1265 and Ser1268) underscores the shared inhibitory mechanism via blockade of the 6‐HB formation critical for viral fusion.

The average RMSD values across the simulated AVP–HR2 complexes ranged from 2.244 to 2.572 Å, offering a measure of structural deviation from the initial complex geometry (Table 5). Lower RMSD values suggest greater conformational stability over the 100 ns simulation. Among the peptides, Griffithsin (2.301 Å), Human Defensin 5 (2.244 Å), and Brevinin‐2 (2.322 Å) displayed the lowest RMSD values, indicating exceptional dynamic stability and minimal deviation during the simulation. In contrast, the standard inhibitor Peptide‐6 (2.572 Å) showed the highest RMSD, suggesting that several natural AVPs may maintain the HR2 complex with greater structural fidelity than the control peptide. The RoG values, reflecting the compactness of the HR2–peptide complexes, were found within a narrow range, from 2.161 Å (Griffithsin) to 2.298 Å (Piscidin 2 and Myticin C). A lower RoG indicates a tighter and more compact complex, which typically correlates with enhanced binding efficiency and reduced solvent exposure. Griffithsin, once again, showed the most compact structure (2.161 Å), followed closely by Brevinin‐2 (2.168 Å), CCL20 (2.171 Å), and hBD‐1 (2.170 Å). These values highlight that these AVPs not only bind stably but also maintain HR2 in a highly compact conformation, a desirable feature for fusion inhibition. On the other hand, HR2 complexes with Piscidin 2 and Myticin C had the highest RoG values (2.298 Å), potentially suggesting less favorable folding or slightly looser binding interactions. Hydrogen bonding is a primary driver of intermolecular stability in peptide–protein interactions. Griffithsin again topped the list with 43 hydrogen bonds, closely followed by Brevinin‐2 (42), CCL20 (40), hBD‐1 (41), and Labyrinthopeptin A2 (39). These peptides formed dense and stable hydrogen‐bonding networks, significantly exceeding that of Peptide‐6, which only formed 24 hydrogen bonds. Such high hydrogen bond counts indicate robust and sustained binding, enhancing inhibitory potential by anchoring the peptide in the HR2 binding pocket throughout the simulation. Several other AVPs also demonstrated strong hydrogen bonding profiles. For instance, Melittin (37), Human Defensin 5 (36), An1a (35), and Latarcin 1 (34) all showed elevated hydrogen bond numbers, indicating promising stability profiles. Conversely, Piscidin 2 (24) and Peptide‐6 (24) had the lowest hydrogen bond counts, potentially reflecting less stable interactions and a more transient binding interface, which may limit their inhibitory effectiveness.

**Table 5:** Time‐averaged structural properties obtained from the MD simulations of AVP‐HR2 complexes.

AVP‐HR2 complex	Average RMSD (Å)	Average RMSF (Å)	Average RoG (Å)	Number of hydrogen bonds between the two proteins
HR2_Peptide‐6 (standard inhibitor)	2.572	0.976	2.282	24
HR2_An1a	2.347	0.942	2.176	35
HR2_Melittin	2.341	0.936	2.174	37
HR2_CCL20	2.330	0.924	2.171	40
HR2_Labyrinthopeptin A2	2.433	0.926	2.172	39
HR2_Lactoferricin B	2.455	0.952	2.181	33
HR2_Griffithsin	2.301	0.888	2.161	43
HR2_Shepherin II	2.378	0.971	2.194	29
HR2_Human neutrophil peptide‐1	2.359	0.956	2.184	32
HR2_Myticin C	2.411	1.115	2.298	33
HR2_Tricyclic peptide RP 71955	2.365	1.021	2.188	30
HR2_Human defensin 5	2.244	0.938	2.176	36
HR2_Human defensin hBD‐1	2.325	0.919	2.170	41
HR2_Neutrophil cationic peptide 1 type B	2.543	1.022	2.187	33
HR2_Brevinin‐2	2.322	0.914	2.168	42
HR2_Latarcin 1	2.351	0.948	2.179	34
HR2_Human neutrophil peptide‐2	2.362	0.959	2.186	31
HR2_Piscidin 2	2.469	1.124	2.298	24
HR2_Varv peptide E	2.495	0.989	2.201	26

The binding free energy (ΔG_binding) obtained from MM/PBSA calculations provides quantitative insight into the binding affinity of AVPs toward the HR2 domain of the MERS‐CoV spike protein (Table 6). Lower (more negative) ΔG_binding values indicate stronger and more thermodynamically favorable interactions, and are used as a benchmark to identify potent inhibitors. In this study, Peptide‐6, a known HR2 inhibitor, served as the standard reference with a ΔG_binding of −49.73 ± 4.08 kcal/mol. This baseline provides a comparative context to assess the relative binding strengths of other candidate AVPs. Among the tested peptides, several demonstrated significantly stronger binding affinities than Peptide‐6. Griffithsin notably exhibited the most favorable binding energy at −213.69 ± 4.73 kcal/mol, suggesting a highly stable complex formation with the HR2 domain. This is particularly interesting given Griffithsin's known antiviral properties, and the strong binding energy implies a potential to lock HR2 into a non‐fusogenic conformation, thereby acting as an effective fusion inhibitor. Similarly, Brevinin‐2 (− 168.83 ± 2.66 kcal/mol), human β‐defensin‐1 (hBD‐1, −166.63 ± 4.15 kcal/mol), CCL20 (− 165.17 ± 2.91 kcal/mol), and Labyrinthopeptin A2 (− 165.13 ± 1.87 kcal/mol) formed a top‐tier group of high‐affinity binders, all outperforming Peptide‐6 by a wide margin. Their strong interaction energies suggest robust molecular engagement with the HR2 domain, which could effectively inhibit membrane fusion events essential for viral entry. Interestingly, Shepherin II (− 106.17 ± 2.59 kcal/mol) and Varv peptide E (− 98.99 ± 4.92 kcal/mol) exhibited comparatively weaker binding among the AVP cohort, although still significantly stronger than Peptide‐6. Their moderate ΔG_binding values suggest that while they may contribute to partial inhibition of HR2, their utility as standalone inhibitors might be limited or require further sequence optimization or chemical modifications to enhance efficacy.

**Table 6:** Top‐performing antiviral peptides (AVPs) targeting the MERS‐CoV HR2 Domain: Binding free energies (ΔG_binding_) from MM/PBSA calculations.

AVP‐HR2 complex	MM/PBSA calculation results ΔG_binding_ (kcal/mol)
HR2_Peptide‐6 (standard inhibitor)	−49.73 ± 4.08
HR2_An1a	−138.97 ± 2.13
HR2_Melittin	−145.99 ± 3.56
HR2_CCL20	−165.17 ± 2.91
HR2_Labyrinthopeptin A2	−165.13 ± 1.87
HR2_Lactoferricin B	−130.55 ± 3.42
HR2_Griffithsin	−213.69 ± 4.73
HR2_Shepherin II	−106.17 ± 2.59
HR2_Human neutrophil peptide‐1	−127.17 ± 1.96
HR2_Myticin C	−154.63 ± 2.77
HR2_Tricyclic peptide RP 71955	−121.32 ± 1.85
HR2_Human defensin 5	−142.05 ± 3.21
HR2_Human defensin hBD‐1	−166.63 ± 4.15
HR2_Neutrophil cationic peptide 1 type B	−153.91 ± 3.94
HR2_Brevinin‐2	−168.83 ± 2.66
HR2_Latarcin 1	−133.42 ± 1.78
HR2_Human neutrophil peptide‐2	−124.60 ± 2.35
HR2_Piscidin 2	−147.04 ± 1.42
HR2_Varv peptide E	−98.99 ± 4.92

### 3.2 Predicted Hemolytic Activity Profiles of AVPs

The hemolytic potential of AVPs is a critical safety parameter when evaluating their candidacy as therapeutic agents. Hemolysis, or the rupture of red blood cells, can lead to toxic side effects and limit the clinical applicability of peptide‐based therapeutics (Yang and Xu 2024). Table 7 presents the predicted hemolytic activity profiles for a panel of AVPs targeting the MERS‐CoV HR2 domain. These predictions are based on hybrid scoring derived from machine learning (ML) classifiers and motif enrichment (MERCI) scores. The hybrid score integrates statistical and pattern‐based features to generate a single probability metric, where a value approaching 1.0 indicates a high risk of hemolytic activity. In contrast, values near 0.0 suggest non‐hemolytic behavior. Among the 20 peptides evaluated, 19 were predicted to be non‐hemolytic, including the standard inhibitor Peptide‐6, which had a hybrid score of 0.0, indicating a very low likelihood of inducing hemolysis. This is consistent with its previous safe usage as a model fusion inhibitor. Most peptides, including Griffithsin, An1a, Melittin, Lactoferricin B, and Labyrinthopeptin A2, also showed hybrid scores of 0.0, despite some of them (like Melittin) having known cytolytic effects in other contexts. This suggests that their sequence‐specific interactions with erythrocyte membranes may be minimal under physiological conditions or in the computational context assessed here. The only peptide flagged as hemolytic was Varv peptide E, which had a hybrid score of 0.742. This value indicates a high likelihood of inducing hemolysis and aligns with its sequence characteristics, which likely include amphipathic and highly cationic domains typical of membrane‐lytic peptides. Despite showing moderate binding affinity in molecular docking and MM/PBSA evaluations, its predicted toxicity poses a significant limitation. Further engineering, such as alanine scanning or residue substitution, may be necessary to mitigate its hemolytic potential while preserving antiviral efficacy.

**Table 7:** Haemolytic activity prediction of selected antiviral peptides (AVPs). The table presented below furnishes information about the user‐input query peptides, encompassing their IDs, pattern names (which signify hemolytic motifs), machine learning scores (indicating the likelihood of toxicity), MERCI scores, hybrid scores (which amalgamate ML and MERCI), and overall predictions (whether they are hemolytic or non‐hemolytic).

Antiviral Peptide	Sequence	ESM Score	MERCI Score	Hybrid Scores	Prediction
Peptide‐6 (standard inhibitor)	SLTQINWTLLDLTYEMESLQQVVKALNEYYIDLKHL	0.675	−1.0	0.0	Non‐Haemolytic
An1a	METAHVFLLSFLLLCVFAVDLIEAGFGCPLDQMQCHNHCQSVRYRGGYCTNFLKMTCKCYG	0.679	−1.0	0.0	Non‐Haemolytic
Melittin	GIGAVLKVLTTGLPALISWIKRKRQQ	0.764	−1.0	0.0	Non‐Haemolytic
CCL20	SNFDCCLGYTDRILHPKFIVGFTRQLANEGCDINAIIFHTKKKLSVCANPKQTWVKYIVRLLSKKVKNM	0.674	−0.5	0.174	Non‐Haemolytic
Labyrinthopeptin A2	MASILELQNLDVEHARGENRSDWSLWECCSTGSLFACC	0.724	−1.0	0.0	Non‐Haemolytic
Lactoferricin B	FKCRRWQWRMKKLGAPSITCVRRAF	0.479	−1.0	0.0	Non‐Haemolytic
Griffithsin	SLTHRKFGGSGGSPFSGLSSIAVRSGSYLDAIIIDGVHHGGSGGNLSPTFTFGSGEYISNMTIRSGDYIDNISFETNMGRRFGPYGGSGGSANTLSNVKVIQINGSAGDYLDSLDIYYEQY	0.636	−1.0	0.0	Non‐Haemolytic
Shepherin II	GYHGGHGGHGGGYNGGGGHGGHGGGYNGGGHHGGGGHG	0.706	−0.5	0.206	Non‐Haemolytic
Human neutrophil peptide‐1	ACYCRIPACIAGERRYGTCIYQGRLWAFCC	0.742	−1.0	0.0	Non‐Haemolytic
Myticin C	MKATILLAVVVAVIVGVQEAQSVACTSYYCSKFCGSAGCSLYGCYLLHPGKICYCLHCSRAESPLALSGSARNVNDKNNEMENSPLMNEVVNLDQEMNMF	0.666	−0.5	0.166	Non‐Haemolytic
Tricyclic peptide RP 71955	CLGIGSCNDFAGCGYAVVCFW	0.755	−1.0	0.0	Non‐Haemolytic
Human defensin 5	ATCYCRTGRCATRESLSGVCEISGRLYRLCCR	0.709	−1.0	0.0	Non‐Haemolytic
Human defensin hBD‐1	DHYNCVSSGGQCLYSACPIFTKIQGTCYRGKAKCCK	0.684	−1.0	0.0	Non‐Haemolytic
Neutrophil cationic peptide 1 type B	RRCICTTRTCRFPYRRLGTCIFQNRVYTFCC	0.729	−1.0	0.0	Non‐Haemolytic
Brevinin‐2	GIWDTIKSMGKVFAGKILQNL	0.722	−1.0	0.0	Non‐Haemolytic
Latarcin 1	SMWSGMWRRKLKKLRNALKKKLKGE	0.221	−1.0	0.0	Non‐Haemolytic
Human neutrophil peptide‐2	CYCRIPACIAGERRYGTCIYQGRLWAFCC	0.741	−1.0	0.0	Non‐Haemolytic
Piscidin 2	MKCATLSLVLSMVVLMAEPGDAFFHHIFRGIVHVGKTIHKLVTGGKAEQDQQDQQYQQDQQDQQAQQYQRFNRERAAFD	0.679	−1.0	0.0	Non‐Haemolytic
Varv peptide E	GLPICGETCVGGTCNTPGCSCSWPVCTRN	0.742	0.0	0.742	Hemolytic

*Note:* A prediction value (hybrid scores) nearing one implies a high likelihood of hemolysis, while a value nearing zero indicates non‐hemolytic properties.

### 3.3 Predicted Physicochemical Properties of AVPs

Table 8 summarizes key parameters such as theoretical isoelectric point (pI), extinction coefficients, estimated *in vitro* half‐lives, instability indices, aliphatic indices, and GRAVY (grand average of hydropathicity) scores, each contributing to a holistic evaluation of peptide performance. The instability index is a widely used predictor of peptide degradation propensity, where values below 40 suggest stable peptides. Based on this metric, Peptide‐6, CCL20, Human defensin 5, Brevinin‐2, and Latarcin 1 exhibit strong stability, supporting their viability for therapeutic application. Conversely, peptides like Lactoferricin B (77.92), Human neutrophil peptide‐1 (55.71), and Labyrinthopeptin A2 (59.12) have high instability scores, suggesting a greater likelihood of rapid degradation unless stabilized via formulation or chemical modification. Estimated half‐lives also vary, with many peptides, such as An1a, Melittin, Myticin C, and Brevinin‐2, demonstrating relatively long half‐lives (up to 30 h), indicating promising pharmacokinetic profiles. In contrast, peptides like Lactoferricin B and Human defensin hBD‐1 have shorter half‐lives around 1–1.2 h, which may necessitate sustained‐release delivery systems for therapeutic use. The GRAVY score reflects the hydropathicity of a peptide, where negative values imply hydrophilicity and likely solubility in aqueous environments—an essential trait for systemic delivery. Peptides like Peptide‐6 (− 0.083), Griffithsin (− 0.240), and CCL20 (− 0.106) fall in this range, indicating a balance between water solubility and potential membrane interaction. On the other hand, Shepherin II (− 1.224) and Latarcin 1 (− 1.248) show highly negative GRAVY scores, suggesting extreme hydrophilicity that could limit membrane permeability or require formulation aids for intracellular access. Meanwhile, highly positive GRAVY values, such as for Tricyclic peptide RP 71955 (1.157), imply hydrophobicity, which can enhance membrane penetration but also raise concerns regarding aggregation or non‐specific toxicity.

**Table 8:** Physicochemical properties of antiviral peptides (AVPs), including stability, hydrophobicity, and half‐life predictions. These parameters were computed using the ProtParam tool, providing insights into each peptide's potential behavior in biological environments.

Antiviral peptide	Theoretical pI	Extinction coefficients*	Estimated half‐life**	Instability index	Aliphatic index	GRAVY***
Peptide‐6 (standard inhibitor)	4.50	9970	1.9 h	16.41	127.22	−0.083
An1a	6.78	4845	30 h	25.56	81.48	0.413
Melittin	12.02	5500	30 h	44.73	135.00	0.273
CCL20	9.70	8730	1.9 h	8.19	93.19	−0.106
Labyrinthopeptin A2	4.35	11,250	30 h	59.12	77.11	−0.084
Lactoferricin B	11.84	11,125	1.1 h	77.92	50.80	−0.576
Griffithsin	5.39	11,920	1.9 h	39.86	70.91	−0.240
Shepherin II	7.28	4470	30 h	27.98	0.00	−1.224
Human neutrophil peptide‐1	8.68	10,345	4.4 h	55.71	65.33	0.300
Myticin C	5.52	7950	30 h	39.38	88.70	0.242
Tricyclic peptide RP 71955	3.80	7240	1.2 h	33.82	74.29	1.157
Human defensin 5	8.96	3355	4.4 h	13.79	64.06	−0.113
Human defensin hBD‐1	8.87	4845	1.1 h	34.49	46.11	−0.272
Neutrophil cationic peptide 1 type B	9.80	3355	1 h	53.98	47.10	−0.200
Brevinin‐2	9.70	5500	30 h	2.80	111.43	0.286
Latarcin 1	11.77	11,000	1.9 h	18.36	66.40	−1.248
Human neutrophil peptide‐2	8.67	10,345	1.2 h	42.13	64.14	0.248
Piscidin 2	6.38	2980	30 h	42.36	70.38	−0.605
Varv peptide E	5.96	5875	30 h	39.95	46.90	0.159

* Extinction coefficients are in units of M^−1^ cm^−^^1^, at 280 nm measured in water.

** Mammalian reticulocytes, *in vitro*.

*** Grand average of hydropathicity.

The aliphatic index estimates a peptide's thermostability, with higher values generally correlating with improved stability under physiological conditions. Melittin (135.00) and Peptide‐6 (127.22) demonstrate high aliphatic indices, indicating their strong thermal resilience and potential for bioactivity across varying conditions. In contrast, Shepherin II (0.00), with no aliphatic side chains, may be more structurally vulnerable in fluctuating environments unless stabilized by cyclization or secondary structure constraints. The theoretical pI values suggest the pH at which the peptide carries no net charge. This affects solubility and interaction with cell membranes or target proteins. Most peptides range from mildly acidic to fundamental pI values. For example, Melittin (pI 12.02) and Lactoferricin B (11.84) are strongly cationic, favoring electrostatic interaction with negatively charged viral membranes or receptors. Conversely, Tricyclic peptide RP 71955 (pI 3.80) and Labyrinthopeptin A2 (4.35) are more acidic, which might influence their biodistribution and target selectivity.

## 4 Discussion

Griffithsin, Brevinin‐2, and CCL20 have emerged as leading AVP candidates targeting the HR2 domain of the MERS‐CoV spike protein. Their potential efficacy has been comprehensively evaluated through an integrated approach involving peptide‐protein docking, MD simulations, hemolytic activity predictions, and detailed assessments of physicochemical properties. These multi‐faceted analyses provide critical insights into their prospective use as therapeutic agents, particularly when compared to the benchmark standard inhibitor, Peptide‐6. The promising antiviral activities of Griffithsin, Brevinin‐2, and CCL20 targeting the MERS‐CoV HR2 domain are strongly supported by a growing body of literature emphasizing the therapeutic potential of HR2‐derived peptides and other fusion inhibitors. Prior studies on HR2P peptides and their engineered analogs have consistently demonstrated their ability to disrupt the 6‐HB formation during the membrane fusion process of coronaviruses (Shuai Xia et al. 2019; Shuai Xia et al. 2019). This 6‐HB structure, formed by the interaction of HR1 and HR2 domains within the viral spike protein, is indispensable for bringing the viral and host membranes into proximity, facilitating viral entry (Santopolo et al. 2021; Zhu et al. 2021). By competitively binding to HR1 or HR2 regions, these peptides effectively prevent the conformational rearrangements necessary for 6‐HB assembly, thereby blocking viral fusion and subsequent infection.

Specifically, HR2P peptides have been experimentally validated to inhibit MERS‐CoV fusion *in vitro*, showing dose‐dependent reductions in viral entry and replication. These peptides also exhibit synergistic effects when combined with other antiviral agents, suggesting potential for combination therapies (Channappanavar et al. 2015). In addition to HR2P analogs, the pan‐coronavirus fusion inhibitor EK1 and its derivatives (e.g., EK1C4) have demonstrated broad‐spectrum activity against MERS‐CoV, SARS‐CoV, and SARS‐CoV‐2 by targeting the conserved HR1 domain and preventing 6‐HB formation (Guo et al. 2023; Xue et al. 2022). Compared with these classical HR2‐derived inhibitors, Griffithsin, Brevinin‐2, and CCL20 exhibit both mechanistic similarities and notable differences. Importantly, the binding free energy values obtained in our MM/PBSA analysis for Griffithsin (− 213.69 kcal/mol), Brevinin‐2 (− 168.83 kcal/mol), and CCL20 (− 165.17 kcal/mol) were substantially more favorable than that of the validated HR2 inhibitor Peptide‐6 (− 49.73 kcal/mol), supporting the hypothesis that these peptides may achieve comparable or enhanced inhibitory activity relative to previously characterized HR2‐derived peptides. While HR2P and EK1 primarily function as competitive inhibitors that directly occupy the HR1 grooves (Pozzi et al. 2023), our structural analyses indicate that Griffithsin, Brevinin‐2, and CCL20 interact strongly with hotspot residues such as Glu1265 and Ser1268 within the HR2 interface, suggesting a fusion‐disruptive mechanism that converges functionally with HR2‐derived peptides but originates from structurally distinct peptide scaffolds.

Griffithsin, a lectin‐derived antiviral peptide isolated from red algae, has garnered significant attention for its broad‐spectrum antiviral properties, including potent activity against diverse coronaviruses such as SARS‐CoV and MERS‐CoV. Its mechanism primarily involves high‐affinity binding to high‐mannose glycans on viral envelope glycoproteins, thereby blocking viral attachment and fusion processes (Bains et al. 2023; Decker et al. 2020; Li et al. 2019). In experimental studies, Griffithsin has demonstrated nanomolar‐level inhibitory activity against several enveloped viruses, including SARS‐CoV‐2, HIV, and hepatitis C virus, with minimal cytotoxicity in mammalian cell lines (A. Bains, K. Fischer, W. Guan, & P. J. LiWang, 2023; Kramzer et al. 2021; Meuleman et al. 2011). Beyond direct viral neutralization, Griffithsin also modulates host immune responses, enhancing antiviral defense pathways without eliciting significant cytotoxicity (Ahan et al. 2022).

Similarly, Brevinin‐2, originally isolated from amphibian skin secretions, exhibits a broad range of antimicrobial activities, including antiviral effects. Studies suggest that Brevinin‐2 disrupts viral envelope integrity and interferes with protein‐protein interactions critical for viral entry (Xiong et al. 2021). Its amphipathic and cationic nature facilitates membrane binding and destabilization, enhancing its inhibitory potential against enveloped viruses (Loffredo et al. 2024). Although direct in vitro data against MERS‐CoV remain limited, Brevinin‐2 has shown inhibitory activity against several enveloped viral models, supporting its membrane‐active antiviral properties (Xiong et al. 2021). In contrast to HR2P and EK1, which are rationally designed fusion inhibitors targeting conserved heptad repeats (Gao et al. 2025), Brevinin‐2 is a naturally occurring membrane‐active peptide. CCL20, a chemokine with inherent antimicrobial and immunomodulatory functions, has also been implicated in antiviral defense. It can recruit immune cells to infection sites and directly inhibit viral replication through mechanisms that may include interference with viral‐host membrane fusion (Bayat et al. 2021; Kallal et al. 2010). Previous in vitro studies have shown that CCL20 exhibits antiviral effects against HIV and influenza viruses, partly through immune cell recruitment and direct antimicrobial activity (Ghosh et al. 2009; Österlund et al. 2005; Wiche Salinas et al. 2021). The alignment of our computational findings with these experimental insights strengthens the validity of Griffithsin, Brevinin‐2, and CCL20 as effective MERS‐CoV fusion inhibitors. The convergence of peptide‐protein binding data, stability assessments, and safety predictions with well‐established literature highlights their translational potential. Moreover, these peptides’ distinct but complementary modes of action provide opportunities for designing multi‐targeted antiviral strategies, potentially overcoming viral resistance mechanisms.

### 4.1 Limitations, Clinical Implications, and Future Works

While the current computational study provides valuable insights into the potential efficacy of Griffithsin, Brevinin‐2, and CCL20 as antiviral peptides targeting the MERS‐CoV HR2 domain, several limitations must be acknowledged. First, the findings are primarily based on *in silico* approaches, including molecular docking, MD simulations, and binding free energy calculations. Although these techniques are powerful for predicting molecular interactions and stability, they cannot fully replicate the complexity of biological systems, such as cellular uptake, peptide degradation, immune system interactions, or pharmacokinetics. Secondly, the predicted half‐lives and hemolytic activity scores are estimations based on physicochemical properties and computational models. Experimental validation through *in vitro* and *in vivo* assays is essential to confirm these safety profiles and stability metrics. Additionally, the peptides' antiviral activity against MERS‐CoV must be corroborated by cell culture infection models and ultimately in animal studies to assess efficacy and toxicity comprehensively. Lastly, the potential for immunogenicity and peptide‐induced off‐target effects remains unknown. As peptides can trigger immune responses or interact with unintended molecular targets, detailed immunotoxicological studies will be necessary before clinical translation. Furthermore, although computational pharmacokinetic (PK) and pharmacodynamic (PD) modeling could provide additional translational insight, such analyses were not incorporated into the present study. Peptide‐based therapeutics frequently exhibit complex absorption, distribution, metabolism, and excretion (ADME) profiles that differ substantially from those of small‐molecule antivirals, and reliable in silico prediction of these properties often requires specialized, peptide‐optimized platforms. The absence of comprehensive PK/PD modeling therefore represents a limitation of this work. Future investigations integrating advanced peptide‐specific ADME and PK/PD prediction tools, alongside experimental validation, will be important to more fully characterize the therapeutic feasibility and clinical potential of these antiviral peptide candidates.

Despite these limitations, identifying Griffithsin, Brevinin‐2, and CCL20 as strong binders to the HR2 domain with favorable safety and stability profiles highlights their potential as novel therapeutic agents against MERS‐CoV. These peptides could be developed as fusion inhibitors, representing a strategic approach to block viral entry at an early stage, reducing viral load and transmission risk. Moreover, Griffithsin's broad‐spectrum antiviral activity suggests potential applicability beyond MERS‐CoV, including other coronaviruses and enveloped viruses, making it a valuable candidate for pandemic preparedness. The relatively favorable physicochemical and safety profiles of Brevinin‐2 and CCL20 further support their possible use in combination therapies, which may mitigate the emergence of resistant viral strains. From a formulation perspective, the peptides’ stability and solubility parameters suggest potential for development as injectable or inhalable therapeutics, targeting respiratory tract infections directly. However, appropriate delivery systems, dosage regimens, and pharmacodynamics require further optimization.

Future research should prioritize experimental validation of Griffithsin, Brevinin‐2, and CCL20 through *in vitro* antiviral assays and *in vivo* efficacy and safety studies to confirm their therapeutic potential. Investigations into their pharmacokinetic and pharmacodynamic profiles will be essential to optimize dosing strategies. Additionally, efforts to enhance peptide stability and bioavailability via structural modifications and the development of targeted delivery systems will be critical for clinical translation. Exploring combination therapies with other antivirals could further improve efficacy and reduce resistance risk. Collectively, these steps will advance these promising peptides from computational candidates to effective antiviral agents against MERS‐CoV and related viruses.

## 5 Conclusions

In conclusion, this study highlights Griffithsin, Brevinin‐2, and CCL20 as promising antiviral peptide candidates targeting the HR2 domain of the MERS‐CoV spike protein. Through comprehensive computational analyses, including molecular docking, molecular dynamics simulations, binding free energy calculations, and physicochemical property assessments, these peptides demonstrated superior binding affinity, stability, and safety profiles compared to the standard inhibitor Peptide‐6. Their ability to disrupt critical HR2 interactions necessary for viral fusion suggests strong potential to inhibit MERS‐CoV entry effectively. While these findings provide a solid foundation for therapeutic development, further experimental validation is essential to confirm antiviral efficacy and safety *in vitro* and *in vivo*. Additionally, future work should focus on optimizing pharmacokinetic properties, minimizing immunogenicity, and developing effective delivery systems to enhance bioavailability and target specificity. Investigating combination therapies with existing antivirals may also improve clinical outcomes and reduce resistance risk. Overall, this work advances our understanding of peptide‐based fusion inhibitors and contributes valuable insights toward the design and development of novel antiviral agents against coronaviruses.

## Author Contributions

**Nasser Alotaiq:** conceptualization, methodology, investigation, writing – original draft, writing – review and editing, supervision, validation, funding acquisition, project administration. **Doni Dermawan:** methodology, data curation, investigation, formal analysis, visualization. **Samir Chtita:** methodology, software, data curation, formal analysis, investigation, validation. All authors have read and approved the final manuscript.

## Ethics Statement

The authors have nothing to report.

## Conflicts of Interest

The authors declare no conflicts of interest.

## Supporting information

Supporting File 1

Supporting File 2

Supporting File 3

## Data Availability

The data that supports the findings of this study are available in the supporting material of this article.
